# New biomaterials for modern dentistry

**DOI:** 10.1186/s12903-023-03531-9

**Published:** 2023-10-29

**Authors:** Artak Heboyan, Francesco Bennardo

**Affiliations:** 1 Department of Prosthodontics, Faculty of Stomatology, Yerevan State Medical University after Mkhitar Heratsi, Yerevan, 0025 Armenia; 2 School of Dentistry, Magna Graecia University of Catanzaro, Catanzaro, Italy

## Abstract

Whilst the appropriate assessment criteria for dental biomaterials is debated, there has been an increasing interest in the use of dental biomaterials for oral rehabilitation. Consequently, a variety of new biomaterials have been introduced in dentistry. To address this issue, BMC Oral Health has launched a Collection on “New biomaterials for modern dentistry”.

Currently, various bio- and nanomaterials are used in the field of dentistry and dental implantology. For protection and restoration of damaged and missing teeth different biomaterials such as metal alloys, dental cements, dental ceramics, polymers are used [1]. For the whitening of discolored teeth carbamide and hydrogen peroxide are used.

All nano- and biomaterials used in dentistry have its flaws and advantages. Nanotechnology helped to improve the quality of materials and widened application spheres. The majority of disadvantages of materials have been overcome by introduction and application of nanoparticles. The nanoparticles, such as graphene, carbon nanotubes, hydroxy apatite, silver, silica, titania, zirconia etc., increase the quality of the various bioproducts by adding different functional groups to them [2]. Titanium dental implants have been used worldwide for almost half a century with a low failure rate [3]. Due to its chemical property and ability to form crystals with inorganic components, hydroxy apatite that covers implant surface can build chemical bonds and provide immediate integration of titanium implants to surrounding tissues.

Nowadays, dental implants are covered with various biocompatible nanocoates, which enhance osseointegration providing long-lasting results. Incorporation of new nano- and biocoatings onto implant surface can increase the effectiveness of osseintegrative process enhancing development of ankyloses during short period of time [4]. Moreover, porous silicon, nanoporous anodic alumina and titania nanotubes are used for development of drug-releasing implants.

A recent review suggests to search for restorative biomaterials with a composition as similar as possible to dental tissues, which not only provoke a lower inflammatory response, but also favor periodontal health [5]. Moreover, the search for better luting cements that, if they fall into the periodontal sulcus, should degrade easily in the oral environment, but simultaneously that do not degrade under the restoration [5, 6].

Zirconia is a white polycrystalline ceramic with exceptional properties [7]. It has improved the esthetics and translucency of prosthetic restorations and also shows promising results, along with a high survival rate, bending and fracture resistance, encouraging prosthetists to use this biomaterial [8]. A great advantage of zirconia-based ceramic biomaterials is that they are chemically inert, showing no cytotoxicity or adverse effects on oral mucosal cells and tissues. This confers less damage, especially in patients who are genetically susceptible to periodontal disease [9].

Nowadays Zirconia is the most used tooth-colored biomaterial, and PEEK can be considered as an alternative to zirconium. The restorations made of zirconia dioxide produced three times more wear of antagonist teeth, higher color stability, compared with restorations fabricated of PEEK. The PEEK restorations showed minimal abrasion, better stress modulation through plastic deformation, which makes it a promising alternative to zirconia crowns [9].

Polyetheretherketone (PEEK) is a high-performance polymer that has demonstrated superior mechanical, esthetic, physical and biocompatible properties with diverse applications in different areas of dentistry [10]. On the one hand, in relation to oral implantology, it has been used in the fabrication of implant abutments; temporary abutments, healing abutments and healing caps. In prosthodontics, it has been used in the fabrication of single crowns or fixed partial dentures of three or more units, either provisional (long-term) or definitive, it has also been implemented in removable prostheses, in the fabrication of complete dentures, occlusal splints and in maxillofacial prostheses [11].

Biochemically, it is a thermoplastic polymer consisting of phenylene (aryl) rings, an ether group (R-O-R) and carbonyl groups (R-O-R). On the one hand, the ether groups (R-O-R) ensure structural flexibility, the ketone groups (R-CO-R) provide rigidity and the phenylene groups are non-reactive [12]. Consequently, these functional groups give PEEK an excellent ability to resist chemical attack, good processability, toughness and high strength. In addition, PEEK has been shown to have good biocompatibility with oral tissues, specifically in processes related to cell adhesion, metabolic activity and in the proinflammatory response. In fact, it has also been shown that PEEK has a higher hydrophobicity and lower surface energy (surface wettability) compared to other ceramic materials and dental alloys, which favors the inhibition of biofilms on complete dentures constructed with this type of biomaterial [13].

Bonding of PEEK and Zirconia copings increases after the use of adhesive. Adhesives increase the surface area and thus the bonding of copings with natural tooth. Tensile bond strength of PEEK is more than Zirconia, but the difference is not significant [14].

Platelet-rich fibrin (PRF), a second generation autologous platelet concentrate, was also studied in medicine and dentistry for regenerative procedures, promote tissue healing by releasing autologous growth factors over time. The combination of PRF and medications seems to enhance the possible fields of application of this autologous biomaterial also as a drug delivery system [15].

We invite authors to submit research papers related to the whole spectrum of bio- and nanomaterials used in dentistry and dental implantology.
